# Risk assessment and spread of the potentially invasive *Ceratitis rosa* Karsch and *Ceratitis quilicii* De Meyer, Mwatawala & Virgilio sp. Nov. using life-cycle simulation models: Implications for phytosanitary measures and management

**DOI:** 10.1371/journal.pone.0189138

**Published:** 2018-01-05

**Authors:** Chrysantus Mbi Tanga, Fathiya Mbarak Khamis, Henri E. Z. Tonnang, Ivan Rwomushana, Gladys Mosomtai, Samira A. Mohamed, Sunday Ekesi

**Affiliations:** 1 International Centre of Insect Physiology and Ecology (ICIPE), GPO, Nairobi, Kenya; 2 International Maize and Wheat Improvement Centre (CIMMYT), ICRAF House, United Nations Avenue, Gigiri, Nairobi, Kenya; University of Thessaly School of Agricultural Sciences, GREECE

## Abstract

Integrative taxonomy has resolved the species status of the potentially invasive *Ceratitis rosa* Karsch into two separate species with distinct ecological requirements: *C*. *rosa* “lowland type” and the newly described species *Ceratitis quilicii* De Meyer, Mwatawala & Virgilio sp. nov. “highland type”. Both species are tephritid pests threatening the production of horticultural crops in Africa and beyond. Studies were carried out by constructing thermal reaction norms for each life stage of both species at constant and fluctuating temperatures. Non-linear functions were fitted to continuously model species development, mortality, longevity and oviposition to establish phenology models that were stochastically simulated to estimate the life table parameters of each species. For spatial analysis of pest risk, three generic risk indices were visualized using the advanced Insect Life Cycle Modeling software. The study revealed that the highest fecundity, intrinsic rate of natural increase and net reproductive rate for *C*. *rosa* and *C*. *quilicii* was at 25 and 30°C, respectively. The resulting model successfully fits the known distribution of *C*. *rosa* and *C*. *quilicii* in Africa and the two Indian Ocean islands of La Réunion and Mauritius. Globally, the model highlights the substantial invasion risk posed by *C*. *rosa* and *C*. *quilicii* to cropping regions in the Americas, Australia, India, China, Southeast Asia, Europe, and West and Central Africa. However, the proportion of the regions predicted to be climatically suitable for both pests is narrower for *C*. *rosa* in comparison with *C*. *quilicii*, suggesting that *C*. *quilicii* will be more tolerant to a wider range of climatic conditions than *C*. *rosa*. This implies that these pests are of significant concern to biosecurity agencies in the uninvaded regions. Therefore, these findings provide important information to enhance monitoring/surveillance and designing pest management strategies to limit the spread and reduce their impact in the invaded range.

## Introduction

Temperature has a strong and direct effect on fruit fly development, reproduction and survival [1,2,3]. Global climate change and variability in temperature has also been attributed to the abundance, distribution and high infestation of fruit flies in different agro-ecological zones [4–6,1,7–9,3]. Temperature influences tephritid fruit fly population growth rates, increase in the number of generations, extension of the development season, changes in geographical distribution, crop–pest synchrony and interspecies interactions [1,10,7–9,3]. Estimating the relationship between temperature and development rate, survival and reproduction is thus important for predicting areas most suitable for insect species establishment. Predicting the potential distribution of fruitfly pests within the context of global climate variability and in particular temperature increase could help governments and growers adapt to changes in pest population by developing and equipping farmers with adequate pest management tools to reduce crop losses [11,10].

Changes in pest distribution and abundance in many agricultural systems have been predicted using various models [12,13]. These models are largely analytical tools that have been used to determine the risks associated with the behavior of agricultural pests under varying climatic scenarios [14,15]. Two distinct modeling approaches have been used for the evaluation, understanding, and prediction, of the dynamics of insect populations in agroecosystems and assessments of phytosanitary risks [16,15]. The first approach is the inductive method utilized to fit biologically relevant climatic stress functions to define species range limits. This approach matches the climate where an organism is normally found within a region to where it has not yet been reported using long-term meteorological data [40–44]. The second is the deductive method, which is used to define parameter values based on direct experimental observations of species responses to experimentally determined climatic factors or to phenological observations (process-based climate response models) [16]. It describes the basic physiological principles of the growth and development of a target insect species. These principles include development time, survival and reproduction [21–22]. This approach uses detailed laboratory experiments that produces life-table parameters and allows the simulation of populations according to real or interpolated data for a given region and time [24]. Although linear degree-day models have long been accepted as a basis for building phenology and population dynamics models [25–27], the nonlinearities at high and low temperatures have made them poor predictors of insect development. Due to nonlinearity at the high and low temperatures, non-linear models have been developed [28–30] that includes stochastic functions to account for variability in development times among individuals within a population [31,32]. Development of such phenology models requires knowledge of lower and upper developmental thresholds as well as data on development for each life stage of the pest [33,23].

In Africa, horticulture is undoubtedly one of the most attractive agricultural sub-sectors because it offers excellent opportunities for food and nutritional security, employment especially for youths and women, agro-processing needs, and also generates much-needed cash income for rural households. Hence, an improvement in horticultural productivity is viewed as a major economic development strategy for many sub-Saharan African (SSA) countries and beyond [34–36]. However, tephritid fruit flies afflict fruit and vegetable production and are recognized globally as the most important threat to horticulture, which limits the potentisl economic and social benefits of this activity [37–39]. Without control, direct damage by fruit fly species has been reported to range between 0–80% depending on the fruit or vegetable variety, location and season [40–47]. In addition to direct losses, indirect losses are associated with quarantine restrictions imposed by the importing countries to prevent entry and establishment of fruit fly pests [36,48]. The Natal fruit fly, *Ceratitis rosa* Karsch, is recognized as one of the most economically important tephritid fruit fly pests in Africa attacking over 90 cultivated host plants [49,50]. *Ceratitis rosa* is largely restricted to eastern and southern Africa, but has successfully invaded the Indian Ocean islands of La Réunion and Mauritius, where it has been reported to out-compete the introduced *C*. *capitata* (Weidemann) and the native *Ceratitis catoirii* Guérin-Mèneville [17–21, 23–25, 51–57].

This work focused on *C*. *rosa*, which was originally thought to be restricted to the coastal region of Kenya [50] until 2001, when Copeland and Wharton [58] reported the first occurrence in the central highlands. Barr et al. [59] then conducted molecular studies on the two populations and suggested the possibility of two forms of *C*. *rosa*. Additional studies by Virgilio et al. [60] later confirmed the observation that the two populations of *C*. *rosa* in Kenya were actually made up of two distinct genotypic clusters identified as R1 and R2 [60–62,3] with largely overlapping geographic ranges. Also, through funding received from the International Atomic Energy Agency (IAEA) Coordinated Research Program (CRP) entitled “Resolution of Cryptic Species Complexes of Tephritid Pests to Overcome Constraints to SIT Application and International Trade”, an integrative approach to resolve the species status within the *Ceratitis* FAR (*Ceratitis fasciventris*, *Ceratitis anonae*, *Ceratitis rosa*) cryptic species complex using larval and adult morphology, wing morphometrics, cuticular hydrocarbons, pheromones, microsatellites, developmental physiology, geographical distribution, sexual compatibility, behavioural and chemoecological studies was conducted [61–63]. The studies concluded that the two *C*. *rosa* types represented two separate species with different ecological requirements: *C*. *rosa*, which has been referred to as “R1”, “the hot type” or “lowland type”, and *C*. *quilicii* sp. nov., referred to as “R2”, “the cold type” or “highland type”) [61,64]. Based on this new nomenclature, known museum specimens of *C*. *rosa* were revisited and taxonomically classified into *C*. *rosa* and *C*. *quilicii*. It is important to note that samples from many localities have not yet been assigned, as such the full distributional range of *C*. *rosa* and *C*. *quilicii* remains unknown (M. De Meyer, personal communication).

Previous studies on *C*. *rosa* revealed that no development occured at low temperatures (< 15°C) but development rate increased with temperature up to an optimal temperature range of 27.9–31.4°C [65,1,3]. Beyond the optimal level, development rates decreased, dropping sharply as temperature approached the thermal limits of development and survival [1,3]. The studies by Tanga et al. [3] clearly demonstrates and support the existence of two genetically distinct populations of *C*. *rosa* (*Ceratitis rosa* Karsch and *Ceratitis quilicii* De Meyer, Mwatawala & Virgilio sp. Nov.) that are divergent in their physiological response to temperature with potential consequent implications in the invasion dynamics of these pest, there is a general lack of information on temperature-driven phenology models for these species, which include a set of functions describing temperature-dependency to determine the pests`life history to forecast their development in Africa and beyond, which would be useful in the development of risk maps. This study was therefore designed to develop temperature-dependent population growth model for *C*. *rosa* and the newly identified species *C*. *quilicii*, and to assess their risk of spread in various agro-ecological zones in Kenya, Africa and globally using life-cycle simulation models: The findings on potentail distribution, spread and impacts of these potentially invasive species may inform geographically-targeted policies in order to prevent new invasions and manage existing ones in Africa and beyond.

## Materials and methods

### Ethical statement

This study was not undertaken in national park or any protected areas. It was carried out in laboratory and open field screen houses using host species: guava (*Psidium guajava* L.) and mango (*Mangifera indica* L.) and potentially invasive tephritid fruit fly species (*C*. *rosa* and *C*. *quilicii*). No specific permission was required for these experiments or collections due to the studies not involving endangered or protected species. This article does not contain any studies with human participants or animals performed by any of the authors.

### Fruit fly cultures

The wild populations of *C*. *rosa* and *C*. *quilicii* from Kenya were reared from infested fallen guava fruits collected at Kibarani, Msambweni district (S 04°19'62.8''; E 039°32'41.1''; 34 m a. s. l), and from infested mango fruits collected at Kithoka, Imenti North district (N 00°05'58.9"; E 037°40'39.5"; 1,425 m a. s. l), respectively. Stock cultures of the wild fruit fly populations from infested fruits were obtained following the methodology described by Rwomushana et al. [66]. The larvae of the two populations from the different host fruits were subsequently transferred into carrot-based artificial diet [67] after 2–3 generations on their natural hosts. Thereafter, *C*. *rosa* and *C*. *quilicii* populations were reared on artificial diets for 3–4 generations before commencement of the experiments. The identity of both colonies were confirmed using morphological characters of the adult males as outlined in De Meyer et al. [61]. The *C*. *rosa* colony was kept at 28 ± 1°C, 50 ± 8% RH and photoperiod of L12: D12, while the *C*. *quilicii* colony was kept at 23 ± 1°C, 65 ± 5% RH and photoperiod of L12: D12. Portable digital thermo-hygrometers were placed inside each of the rearing rooms of both fruit fly species to monitor temperature and relative humidity. To maintain the genetic vigour of the insects, about 300–500 new individuals collected from the same districts described above for each species were introduced into the respective colonies every six months. Both colonies were maintained at the Animal Rearing and Containment Unit (ARCU) of the International Centre of Insect Physiology and Ecology (*icipe*), Nairobi, Kenya.

### Egg collection

Newly emerged adults of *C*. *rosa* and *C*. *quilicii* were held separately in well ventilated transparent Perspex rearing cages (30 cm length x 30 cm width x 30 cm height). Adult flies were fed a diet consisting of 3 parts sugar and 1 part enzymatic yeast hydrolysate ultrapure (USB Corporation, Cleveland, OH). Water was also provided on pumice granules. The eggs of each fruit fly species were collected by offering them an oviposition substrate, which consisted of mango fruit domes (fruit skin that had the seed and pulp scooped out). The fruit domes were pierced with entomological pins (0.8 mm diameter) to facilitate oviposition by females. Freshly laid eggs were collected within a uniform time interval of 1 h after oviposition using a moistened fine camel’s hair brush.

### Effect of temperature on development and survival of eggs, larvae and pupae of *C*. *rosa* and *C*. *quilicii*

The experiments were conducted in thermostatically controlled environmental chambers (MIR-554-PE, Sanyo/Panasonic cooled incubators, Japan) set at seven constant temperatures of 10, 15, 20, 25, 30, 33 and 35°C (± 0.03°C), 65 ± 5% RH and 12:12 L:D photoperiod to assess the effects of temperature on the development and survival of eggs, larvae, pupae and adults.

#### Egg

One hundred (100) eggs (1 h old) were randomly selected using a fine camel’s hair brush, counted and carefully lined on moistened sterilized black cloth, which were thereafter placed on top of ≈ 60 g of artificial larval diet inside a Petri dish. The Petri dishes were immediately transferred into the seven controlled environmental chambers. The eggs were observed at 6-hourly intervals and the total number of eggs that had hatched at each temperature regime was recorded under a binocular microscope to determine the time and percentage egg hatch. Egg development time and survival for each replicate were estimated. The experiments were replicated five times. The required temperatures inside the incubators were regularly monitored using standard thermo-hygrometers and experiments in which temperatures fluctuated more than ± 0.03°C were discarded and not included in the analysis.

#### Larva

One hundred neonate larvae (~1 h old) were randomly obtained from the fruit fly cultures and carefully transferred onto filter paper squares (2 cm^2^). The square filter paper containing neonate larvae were placed on top of a 150 g carrot-based artificial diet in 90-mm diameter Petri dishes. The Petri dishes containing the neonates were then placed in rectangular plastic rearing containers (21.5 cm x 15 cm) carrying a thin layer (~ 0.5 cm) of sterilized sand at the bottom for pupation, and then transferred to the thermostatically controlled environmental chambers. The lid of the plastic container (16.5 cm x 11 cm) was screened with cloth netting with a mesh size of 1.3 x 1.3 mm for ventilation. Larvae fed *ad libitum*, and mature larvae were allowed to freely leave the Petri dish into rectangular plastic containers for pupation. The sand was examined daily for newly formed pupae, and puparia were separated from the pupation medium by gentle sifting. Records of larval developmental durations were kept for the two fly populations at each temperature regime. The experiments were replicated five times.

#### Pupa

One hundred newly formed pupae (~ 1 h old) were randomly obtained from the fruit fly stock cultures and kept at the different rearing conditions described previously. Pupae used were placed in Petri dishes (8.6 cm diameter) and transferred into aerated transparent Perspex cages (30 cm x 30 cm x 30 cm) to allow for adult emergence. The cages were monitored on a daily basis for adult emergence, and pupal developmental time and survival were recorded. Those that did not emerge were observed for a longer time (~ 1 month) and recorded as dead in the absence of fly emergence. Number of adult flies that emerged and their sex were recorded daily. The experiments were replicated 5 times.

### Effect of temperature on fecundity, oviposition and longevity of adults

On the day of emergence, one female and one male (~1 h old) were paired and placed individually in aerated transparent Perspex cages (15 x 15 x 10 cm). Adult flies were provided with water on pumice granules and fed on a diet consisting of a mixture of enzymatic yeast hydrolysate powder and sugar in a ratio of 3:1. Thereafter, they were provided with oviposition substrates that consisted of a small mango dome placed over a sterile Petri dish (60 mm x 15 mm) lined with moistened filter paper. Each dome was pierced with an entomological pin to facilitate oviposition. Cotton wool soaked in water was placed in the rearing cages to maintain the relative humidity. The domes were exposed for 24 hr and thereafter the eggs were counted and the survival time of individual adult flies was also recorded. Eggs were collected daily using a moistened fine camel’s hair brush and total number of eggs produced per day over the lifetime of each fly was determined. A total of 15 pairs of adult flies were observed at each tested temperature regime for each fruit fly population.

Similarly, an additional cohort of 100 freshly emerged adult females and 100 males (~1 h old) of each fruit fly population were kept in transparent Perspex cages (30 by 30 by 30 cm; length by width by height) at the different temperature regimes and their longevity and survival time were recorded separately for males and females.

### Field experiments under fluctuating temperatures

Life table experiments were carried out for the fruit fly species (*C*. *rosa* and *C*. *quilicii*) under natural fluctuating temperatures to evaluate if the data collected in the laboratory under constant temperatures provided realistic predictions for development and survival. The experimental cages were maintained on table surfaces (324 cm length x 87 cm width x 76 cm height) in a shaded outdoor location at the *icipe* Duduville campus, Nairobi [S 01° 13' 14.6''; E 036° 53' 44.5'', 1612 m above sea level (a.s.l)], where they received ample light and air ciculation but no direct sunlight. The edges of the tables as well as the feet on which the cages were placed were smeared with Tanglefoot® insect barrier paste (Tangle-trap; The Tanglefoot Company, Grand Rapids, MI) to prevent attack by predacious insect species. The table stands were also placed in containers filled with soapy water to further deter predatory insects from accessing the fruit flies in the cages. The water in the containers was replenished when necessary.

The protocol used was similar to that described above under constant temperatures. Freshly laid eggs (~1 h old) were collected from the rearing cages, counted and distributed in five replicates of 100 eggs each. The eggs were placed in the open air where they were observed daily and the developmental duration of each egg and the total number of eggs that hatched was recorded. For the larval development, 100 newly hatched larvae (~1 h old) were transferred individually with a camel hair brush into a 9-cm-diameter Petri dish containing artificial diet and placed in the open air where they were observed daily and developmental time recorded. The experiments were replicated 5 times. On the other hand, 100 newly developed puparia from the stock cultures (~1 h old) were also randomly selected and placed into Petri dishes before transferring into small transparent Perspex cages (20 length x 20 width x 20 cm heights), where they were observed for emergence. The experiments were replicated 5 times. Daily minimum and maximum temperature was recorded throughout the year at the experimental site using a HOBO UX100 Temp/RH Data Logger (Part # UX100-003).

### Model building and validation using Insect Life Cycle Modeling (ILCYM version 3.0)

The *C*. *rosa* and *C*. *quilicii* phenological models were built using Insect Life Cycle Modeling software (ILCYM version 3.0) [68,15]. The software has tools for building process-based population models for insect species. The model builder uses the same shape distribution approach combined with a rate summation and cohort up-dating for simulation of population dynamic models. ILCYM has several non-linear functions that describe the temperature-dependency of different processes in the life history of insects. These include the development time and its variation between individuals in a population, mortality in each immature life-stage, adult longevity (senescence), and reproduction frequencies of the adults according to temperature. The model builder, using its inbuilt statistical criteria, combined with knowledge on the biology of the species under investigation, facilitates the selection of the best fitting functions for describing these temperature-driven processes and then compiles the processes into a phenological model for each of the populations under investigation [69].

### Temperature-dependent process models and statistical analysis

The relationship between temperature-dependence of different processes in the *C*. *rosa* and *C*. *quilicii* life history and different temperature regimes were analyzed by various non-linear models, using the ILCYM software [69]. The statistical analysis implemented in this software selected the best-fitting model to quantify the effect of temperature on development time, mortality, senescence and reproduction according to inbuilt model selection criteria. These criteria were the Akaike’s Information Criterion (AIC), which defines the goodness of an estimated statistical model, and the coefficient of determination, R^2^, which explains how the models capture the variability within the data. A female ratio of 0.5 was assigned for all the temperature regimes that were studied.

### Modeling development time and distribution of *C*. *rosa* and *C*. *quilicii*

The cumulative probability distribution of *C*. *rosa* and *C*. *quilicii* development time under different temperatures were estimated and normalized. Frequency distributions for insect development time are usually skewed towards longer times and it is assumed that development times of insects at different temperatures are of the same shape. The log-transformed data were arranged into a frequency distribution and fitted to a logit model [22]. The fitted generalized linear models to the normalized development time were the logit model for the egg, larva and pupa with the mathematical expression given as

$$F(x)=\frac{1}{1+\exp\;\left(-(a_{i}+bln\;x)\right)}$$

Where *F*(*x*) is the probability to complete development at time *x*, *lnx* is the natural logarithm of the development days observed, *a* is the intercept corresponding to the temperature (*i*) and *b* is the common slope of the regression model representing the dispersion of the development time in the life stages.

### Development rate

According to Khadioli et al. [70], temperature-dependent development of insects from one stage to another does not follow a linear relationship; as such they are usually less preferred for use in phenology models. Therefore, in describing the relationship between temperature and the development rate of the two fruit fly populations, ILCYM provides several non-linear functions and other models that have been used successfully for many insect species [68]. The development time *d* was used to calculate development rates. The temperature-dependent development rate of *C*. *rosa* and *C*. *quilicii* was best described by the Brière 1 model for all temperature regimes [3] and allowed for the estimation of the upper and lower developmental thresholds [30]. The Brière -1 model is given by the expression:

$$r(T)=aT(T-T_{min})\;X\;\sqrt{T_{max}-T}$$

where, *r* is the developmental rate as a function of temperature (*T*), and ‘*a’* is an empirical constant. The following equation from Brière et al. [30] was used to calculate the optimum temperature:

$$T_{opt}=\frac{4Tmax+3Tmin+\sqrt{{16Tmax}^{2}+{9Tmin}^{2}-16TminTmax}}{10}$$

The mean values for *Tmin*, *T*_*opt*_, and *Tmax* were determined for each life stage of each group of *C*. *rosa* and *C*. *quilicii* using the results generated by the developmental rate models. The choice of the best-fitting function in ILCYM was done using the R^2^ statistics or the AIC [71]. The lower development threshold which is defined as the temperature below which there is no measurable development was estimated by the linear regression.

r(T)=a+b.T

where *r*(*T*) is the development rate, *a* and *b* represent the intercept and the slope of the equation, respectively.

### Immature mortality

The mortality rate in the immature life stage was calculated from the relative frequency of cohort survivors. A polynomial mathematical expression was fitted to describe the relationship between the mortality rate and the temperature for each life stage. The equation is given below

where *M*(*T*) is the rate of mortality at temperature *T*; and *b*_*i*_*s* are parameters to be estimated.

$$M(T)={exp}^{b_{1}+b_{2}x+b_{3}x^{2}}$$

### Adult life span and aging

The mean survival time of the adult was determined for both sexes and the inverse of the survival time was plotted against temperature. A modified four parameter Stinner model [26] was fitted to determine the relationship between adult longevity rate of both the male and female adults and temperature. The mathematical expression of the model is given as

$$S(T)=\frac{C_{1}}{1+e^{\left(k_{1}+K_{2}T\right)}}+\frac{C_{2}}{1+e^{k_{1}+k_{2}(T_{0}-T)}}$$

where, *S*(*T*) is the senescence rate at temperature *T*(°C), *C*_1_ and *C*_2_ are maximum and minimum temperatures, respectively. *T*_*o*_ is the optimum temperature and *K*_1_ and *K*_2_ are constants.

### Temperature-dependent reproduction

For *C*. *rosa*, a polynomial regression was applied to determine the effect of the temperature on the total number of eggs laid per female during her entire life span. The expression of the model is given as

$$F(T)=b_{1}+b_{2}.x+b_{3}.x^{2}$$

where *F*(*T*) is the total eggs per female.

A Gaussian equation [72] was applied to determine the effect of temperature on the total number of eggs laid per *C*. *quilicii* adult female fly. The expression of the model is:

$$f(T)=\frac{1}{1+R_{max}.{e^{-a}(\frac{T-T_{max}}{b})}^{2}}$$

where, *f*(*T*) is the fecundity at temperature *T*(°C), *R*_*max*_ is the maximum fecundity, *T*_*max*_ is the temperature at which maximum fecundity occurs, and *a* and *b* are the fitted parameters representing intercept and slope of the equation, respectively.

An exponential model was fitted to describe the age-specific fecundity rate at each of the test temperatures. The cumulative oviposition rate was plotted against normalized female age expressed as ratio of age in days divided by mean survival time. Below is the formula of the exponential equation which was used for assessing the relative oviposition frequency of both fruit fly species:

$${y=1-e}^{-(aX+bX^{2}+cX^{3})}$$

where, *y* is the cumulative oviposition frequency, *X* is the normalized age of female expressed as a ratio of age in days and mean survival time, and *a*, *b* and *c* are the equation parameters.

### Life table parameters of *C*. *rosa* and *C*. *quilicii*

Using ‘stochastic simulation tool’ in ILCYM, the life table parameters i.e. gross reproductive rate (GRR), net reproductive rate (R_0_), mean generation time (T), intrinsic rate of natural increase (*r*_m_), finite rate of increase (λ), and doubling time (D_t_) of *C*. *rosa* and *C*. *quilicii* were estimated based on the developed phenology model. The simulation method was based on the rate summation and cohort updating approaches (i.e. random determination for the survival and development of each individual to the next stage) [21]. The simulations started with an initial number of 100 individuals from the egg stage for a given constant temperature regime and were performed over seven constant temperatures ranging from 10 to 35°C with 50 repetitions for each temperature. The estimated life table parameters were plotted against the respective temperatures and fitted to a quadratic equation.

$$L_{p}(T)=a+bT+cT^{2}$$

Where, *L*_*p*_(*T*) represents the respective life table parameters (GRR, R_o_, T, r_m_, λ, D_t_) at temperature T (°C), and *a*, *b* and *c* are the model parameters.

### Evaluation of the phenological models

The input data for the validation model were obtained from the experiment conducted outdoors using open field cages under fluctuating temperatures. Data obtained from the temperature and relative humidity data loggers showed that the minimum and maximum temperatures recorded during the experiment period (16^th^ September 2014 to 28^th^ December 2015) ranged between 9.14–16.68°C and 18.84–37.66°C, respectively. Experimental life table data points collected based on developmental duration, mortality, longevity and population growth parameters obtained from fluctuating temperature studies were used for validating the results of the model outputs produced from the laboratory constant temperatures experiments using stochastic simulation. ILCYM stochastically simulates a user-defined number of life tables, each with a user-defined number of individuals, through rate summation and random determination for survival of each individual, and development to the next stage. The output of the simulation is then presented in a graphical format. The Euclidian distance between the observed and stimulated data points is provided to show the difference between the simulated and the observed data points. The closer the Euclidian distance value is to zero (0), the better the developed phenology model at constant temperature and results can be used to predict the species distribution and population abundance in natural field conditions under fluctuating temperatures.

### Climate data used in spatial analysis

The mean minimum and maximum temperature information collected during the outdoor experiment under fluctuating temperature were considered as current climatic conditions. For Kenya, a spatial resolution of 2.5 arc-min was used whereas 10.0 arc-min was applied for Africa. In predicting *C*. *rosa* and *C*. *quilicii* response to climate change, we down-scaled, bias-corrected and open source spatial database accessible at AFRICLIM (http://www.york.ac.uk/environment/research/kite/resources/) to project temperature changes. With validation of the predictive ability of the model, we projected the model worldwide to identify areas of potential distribution and possible invasion for each of the species. The simulation for the global prediction was carried out using the climate data obtained from WorldClim (http://www.worldclim.org/) and CCAFS (http://www.ccafs-climate.org) databases as described in Kroschel et al. [15]. The available downscaled values of current temperature (mean daily minimum and maximum) in raster format were loaded and opened in Quantum Geographic Information System software (QGIS) [73].

### Potential population distribution and risk mapping of *C*. *rosa* and *C*. *quilicii*

The Insect Life Cycle Modeling software (ILCYM version 3.0) [15] was used to generate *C*. *rosa* and *C*. *quilicii* risk maps using the validated phenology model. Linked with GIS, the model allows the simulation of pest risk indices. The indices used for mapping included establishment risk index (ERI), generation index (GI) and activity index (AI), which were produced from the obtained population growth parameters as described by Kroschel et al. [15]. The ERI identifies the area in which the pest may survive and become established and it is estimated based on a daily scale by the following expression:

$$ERI=\frac{\sum_{1}^{i=365}I_{i}}{I_{I}}*net-reproduction$$

Where, *I*_*i*_ is the interval of day *i* (with *i* = 1, 2, 3, ……, 365) and the total number of intervals, *I*_*I*_, is 365. The index is 1 when all the immature stages of *C*. *rosa* or *C*. *quilicii* survive throughout the year at varied proportions, with ERI<1 characterizing areas in which survival and establishment of the population is restricted to certain periods in the year.

The GI estimates the mean number of generations that may be produced within a given year, calculated by averaging sum of the estimated generation lengths in each Julian day as shown in the formula:

$$GI=\frac{\sum_{X=1}^{365}365/Tx}{365}$$

where, *Tx* is the predicted generation length in days at Julian day *x* (*x* = 1, 2, 3, …, 365). This implies that an increase in temperature will leads to an increase in the number of generations per year. But, in real life, extreme temperatures reduce fecundity and increase mortality, disrupting the finite rate of natural increase (λ).

The AI is explicitly related to the finite rate of population increase of *C*. *rosa* and *C*. *quilicii* populations, which takes the whole life history of the pest into consideration. The index is calculated by taking a log of products of the estimated finite rates of natural increase for each Julian day as shown below:

$$AI={log}_{10}\prod_{X=1}^{365}\lambda_{X}$$

where, λ_*x*_ is the finite rate of increase at Julian day *x* (*x* = 1, 2, 3, …, 365). Every increase of AI by 1 implies a 10-fold increase of the pest population [24].

Using the index interpolator (a sub-module of ILCYM), the compiled *C*. *rosa* and *C*. *quilicii* phenology, the Digital Elevation Model (DEM) obtained from the Shuttle Radar Topography Mission (SRTM), and the temperature data in text files, were imputed into ILCYM and the pest risk indices generated in American Standard Code for Information Interchange (ASCII) formats [74]. Obtained results were transferred into QGIS for analysis, visualization and interpretation.

### Statistical analysis and modeling tools

All analyses for developing the temperature-dependent phenology model were conducted using the ‘model builder’ tool in Insect Life Cycle Modelling (ILCYM) software, version 3.0 (International Potato Centre, Lima, Peru) [69]. The best fit model in each case was selected based on well-known goodness of fit indicators such as Akaike’s Information Criterion (AIC) [71] and coefficient of determination (R^2^) along with application of our expert knowledge on biology of *C*. *rosa* and to predict their life history under a range of environmental temperatures. We then used Analysis of Variance (ANOVA) and least significance difference (LSD) post-hoc tests at a significance level of P = 0.05 for probability thresholds and hypothesis testing in all the regressions to determine which individual traits were governed by these factors.

## Results

### Temperature-dependent mortality rates of *C*. *rosa* and *C*. *quilicii*

**Fig 1:**
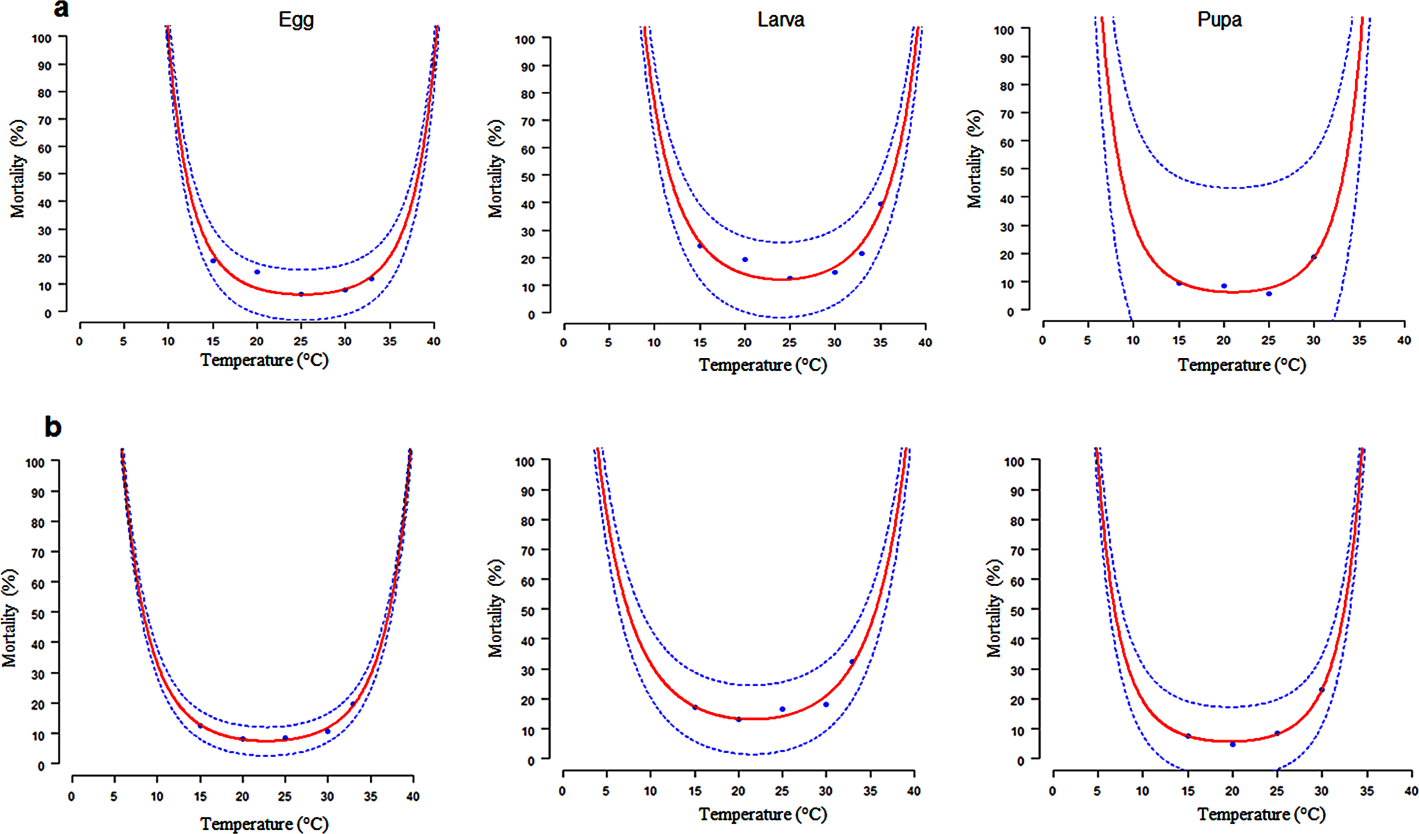
Temperature-dependent mortality rates of *C*. *rosa* (1a) and *C*. *quilicii* (1b) immature life stages (egg; larva and pupa) at six different constant temperatures. Fitted curves: Polynomial regression for all immature stages. The upper and lower 95% confidence intervals of the model are indicated. Markers are observed means, bars represent standard deviation.

The lethal time period when mortality reached 50% (LT 50) or 100% (LT 100) varied considerably between *C*. *quilicii* (Fig 1A and Table 1) and *C*. *rosa* (Fig 1B and Table 1) immature life stages (egg; larva and pupa). According to the model, *C*. *quilicii* had a lower LT100 threshold at 5.2, 4.8 and 5.9°C and an upper LT100 threshold at 35.2, 35.1 and 33.3°C for the eggs, larvae and pupae, respectively. For *C*. *rosa* the lower LT100 was observed at 10.4, 9.6 and 7.2°C, and an upper LT100 threshold at 37.6, 37.8 and 33.3°C for the eggs, larvae and pupae, respectively. The LT50 of *C*. *quilicii* was recorded at 25°C for eggs and larvae, while that of the pupae was 23.8°C. The LT50 of *C*. *rosa* was recorded at 23.6°C for the eggs and larvae, while that of the pupa was at 20°C.

**Table 1:** Estimated parameters (mean ± SE) of the exponential model: *f*(*T*) = *exp* (*b*1 + *b*2.*x* + *b*3.*x*^2^; fitted to mortality rate for eggs, larva and pupa stages of *Ceratitis rosa* and *Ceratitis quilicii*.

	Parameters	Immature life stage		
		Eggs	Larva	Pupa
*C*. *rosa*	b1	10.271 ± 0.002	9.621 ± 0.0014	7.178 ± 0.0031
	b2	-0.909 ± 0.0235	-0.453 ± 0.0161	-0.570 ± 0.0332
	b3	0.019 ± 0.0006	0.009 ± 0.0005	0.014 ± 0.0012
	*r*^*2*^	0.984	0.881	0.912
*C*. *quilicii*	b1	5.183 ± 0.0008	4.812 ± 0.0012	5.8716 ± 0.0560
	b2	-0.422 ± 0.0086	-0.290 ± 0.0140	0.3892 ± 0.0020
	b3	0.009 ± 0.0003	0.007 ± 0.0005	141.781 ± 0.0054
	*r*^*2*^	0.973	0.936	0.912

### Adult reproduction and longevity of *C*. *rosa* and *C*. *quilicii*

The preoviposition, oviposition and postoviposition duration of both *C*. *rosa* and *C*. *quilicii* were inversely correlated with temperature (Table 2). The preoviposition, oviposition and post-oviposition durations were significantly different across the different temperature regimes for *C*. *rosa* and *C*. *quilicii*. At the same temperature, the pre-oviposition duration for *C*. *rosa* and *C*. *quilicii* was significantly different at 15°C, 20°C and 33°C, whereas it was comparable at 25°C, 30°C and 35°C. The oviposition duration of *C*. *rosa* and *C*. *quilicii* was significantly different at 15°C, 20°C, 30°C and 35°C, but comparable at 25°C and 33°C. On the other hand, the post-oviposition duration of *C*. *rosa* and *C*. *quilicii* was significantly different at 25°C, 33°C and 35°C, while it was similar at 15°C, 20°C and 30°C for both fruit flies. However, there were significant interactions between the different temperature regimes investigated and fruit fly species with regards to pre-oviposition (F = 5.54; d.f. = 5, 168; P = 9.45x10^-05^), oviposition (F = 6.78; d.f. = 5, 168; P = 8.8x10^-06^) and post-oviposition duration (F = 4.09; d.f. = 5, 168; P = 1.57x10^-03^).

**Table 2:** Effect of different constant temperature on the pre-oviposition, oviposition, post-oviposition periods (days), fecundity and longevity of *Ceratitis rosa* and *Ceratitis quilicii*.

Temp (°C)	Pre-oviposition		Oviposition		Post-oviposition		Fecundity	
	*C*. *rosa*	*C*. *quilicii*	*C*. *rosa*	*C*. *quilicii*	*C*. *rosa*	*C*. *quilicii*	*C*. *rosa*	*C*. *quilicii*
15	58.27±3.48^a^B	43.27±2.86^a^A	79.27±3.70^a^B	92.87±4.09^a^A	48.33±2.45^a^A	55.33±5.51^a^A	153.2±12.73^c^B	242.13±19.42^d^A
20	28.93±1.87^b^B	23.07±1.71^b^A	67.27±1.91^b^B	88.87±4.65^a^A	17.87±1.34^b^A	20.47±3.31^b^A	297.87±28.61^c^B	402.07±35.93^c^A
25	14.53±1.20^c^A	17.87±1.43^c^A	52.13±3.19^c^A	46.27±2.72^b^A	12.53±1.19^c^B	7.27±2.01^c^A	776.07±43.19^a^B	617.13±39.62^a^A
30	12.47±1.16^c^A	15.33±1.31^cd^A	35.13±2.31^d^B	28.67±2.21^c^A	8.13±1.22^d^A	5.47±1.16 ^c^A	805.73±79.86^a^A	523.67±35.16^b^B
33	10.33±0.54^c^B	12.07±0.58^d^A	24.27±2.05^e^A	21.73±1.55^c^A	6.53±0.61 ^d^B	3.67±1.22 ^c^A	625.47±49.16^b^B	393.27±20.83^c^A
35	9.73±0.38^c^A	11.07±0.69^d^A	18.27±1.32^e^B	12.73±0.92^d^A	3.73±0.52 ^d^B	2.27±0.82 ^c^A	179.13±13.90^c^B	118.47±8.90^e^A

Means in the same column followed by the same upper case and in the same row followed by the same lower case letter are not significantly different [ANOVA and Student–Newman–Keul’s (SNK) test, P < 0.05].

**Fig 2:**
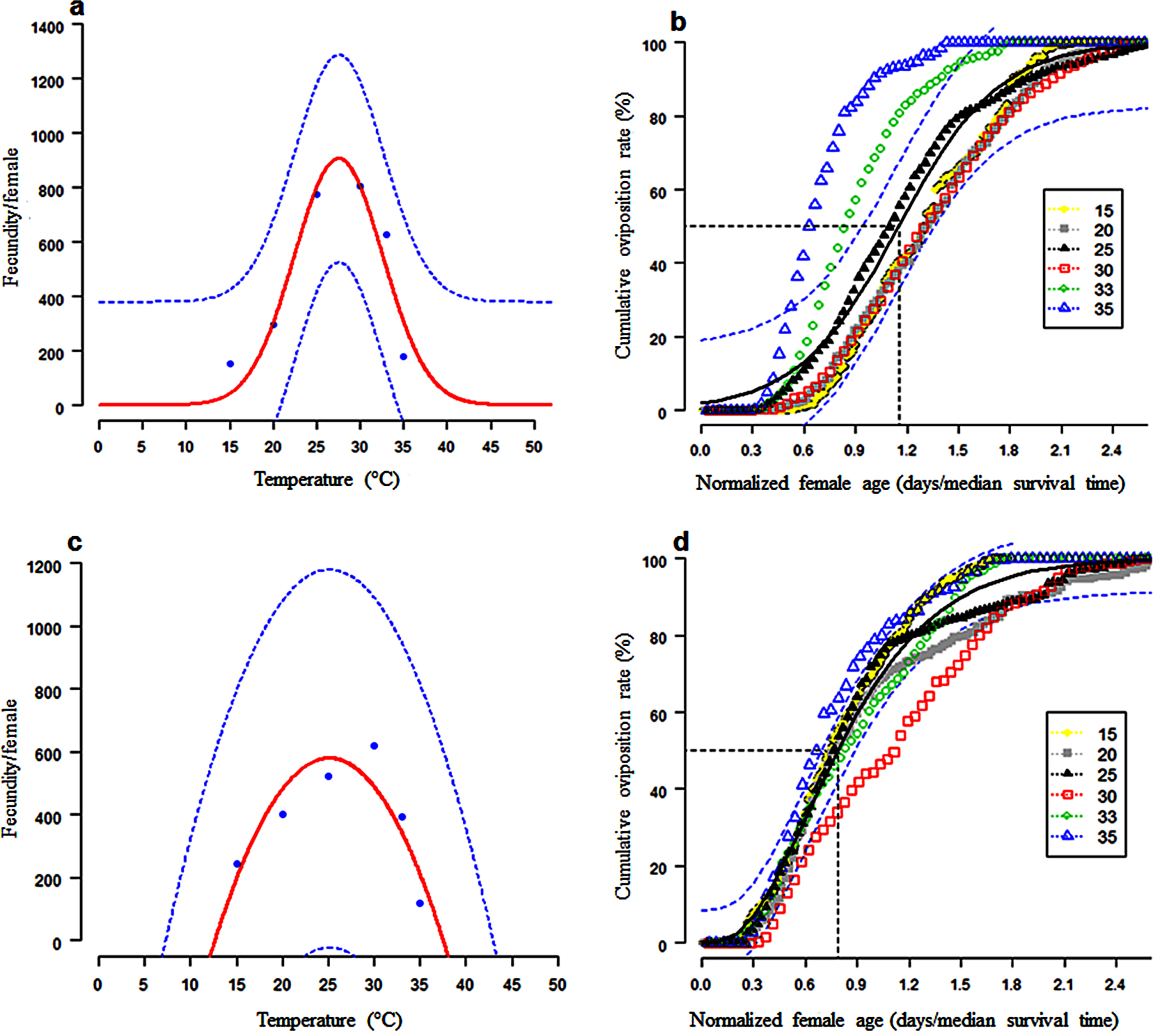
Temperature-dependent total egg production curve for *C*. *rosa* (fitted function: Polynonmial model) and *C*. *quilicii* (fitted function: Guassian denomination function) (2a and 2c, respectively). Age-related oviposition rate for *C*. *rosa* (2b; fitted curve: Second-order polynomial function) and *C*. *quilicii* (2d; fitted curve: Gamma distribution function). The dots are observed data points at each of the test temperatures. The upper and lower 95% confidence intervals of the models are indicated. Age of the females at 50% oviposition is indicated.

The mean number of eggs produced by *C*. *rosa* and *C*. *quilicii* throughout their entire life span was found to vary significantly across the different temperature regimes. Within the same temperature regime the lifetime fecundity of *C*. *rosa* and *C*. *quilicii* was found to vary significantly. Similarly, the interactions between the two fruit fly species and the temperature regimes were found to be highly significant for adult fecundity (F = 11.85; d.f. = 5, 168; P = 7.97x10^-10^). The lifetime fecundity per adult female of *C*. *rosa* was found to range between 153.2 ± 12.7 eggs at 15°C to 805.7 ±79.9 at 30°C, while for *C*. *quilicii*, it ranged between 118.5 ± 8.9 eggs at 35°C to 617.1 ± 39.6 eggs at 25°C (Table 2). The effects of temperature on the fecundity of *C*. *rosa* was described by a second-order polynomial function (F = 13.98; d.f. = 2, 5; P = 0.0302) (Fig 2A and Table 3), while the cumulative proportion of eggs produced per female and normalized female age was described by a second-order modified exponential function (Fig 2B and Table 3). For *C*. *quilicii*, the effects of temperature on fecundity was described by a simple Guassian function (F = 2.15; d.f. = 2, 5; P = 0.3333) (Fig 2C and Table 4), while the cumulative proportion of eggs produced per female and normalized female age was well described by the gamma-function (Fig 2D and Table 4).

**Table 3:** Relationship of the total eggs per *Ceratitis rosa* female with temperature (polynomial function), and age-related cumulative proportion of progeny produced (exponential function).

Response variable	Second-order polynomial function: *f*(*T*) = *exp*(*b*1 + *b*2.*x* + *b*3.*x*^2^			
	*b1*	*b2*	*b3*	*r*^*2*^
Total eggs/female	-7.851 (0.0019*)	1.064 (0.0258*)	-0.019 (0.0009*)	0.903
	Second-order exponential modified function: O(E) = 1/(1+exp(a+bE))			
	*a*	*b*		*r*^*2*^
Proportion of progeny production	3.94859 (0.0933*)	-3.43037 (0.0778*)		0.9399

Numbers in parentheses are standard errors of estimates

*f(T), fecundity at temperature T (°C).

**Table 4:** Relationship of the total eggs per *Ceratitis quilicii* female with temperature (Guassian denomination function), and age-related cumulative proportion of progeny produced (Gamma function).

Response variable	Gaussian denomination function: *f*(*T*) = *y*0 + *a*.*exp*(−0.5((*x* − *x*0)/*b*)^2^)				
	*y0*	*a*	*b*	*x0*	*r*^*2*^
Total eggs/female	- 41393.36 (3.8096*)	41971.86 (29.3741*)	74.63 (19.0522*)	25.126 (7.8725*)	0.7632
	Gamma function: O(E) = pgamma (E,a,b)				
		*a*	*b*		*r*^*2*^
Proportion of progeny production		3.404 (0.0686*)	3.906 (0.08242*)		0.9823

Numbers in parentheses are standard errors of estimates

*f(T), fecundity at temperature T (°C)

**Fig 3:**
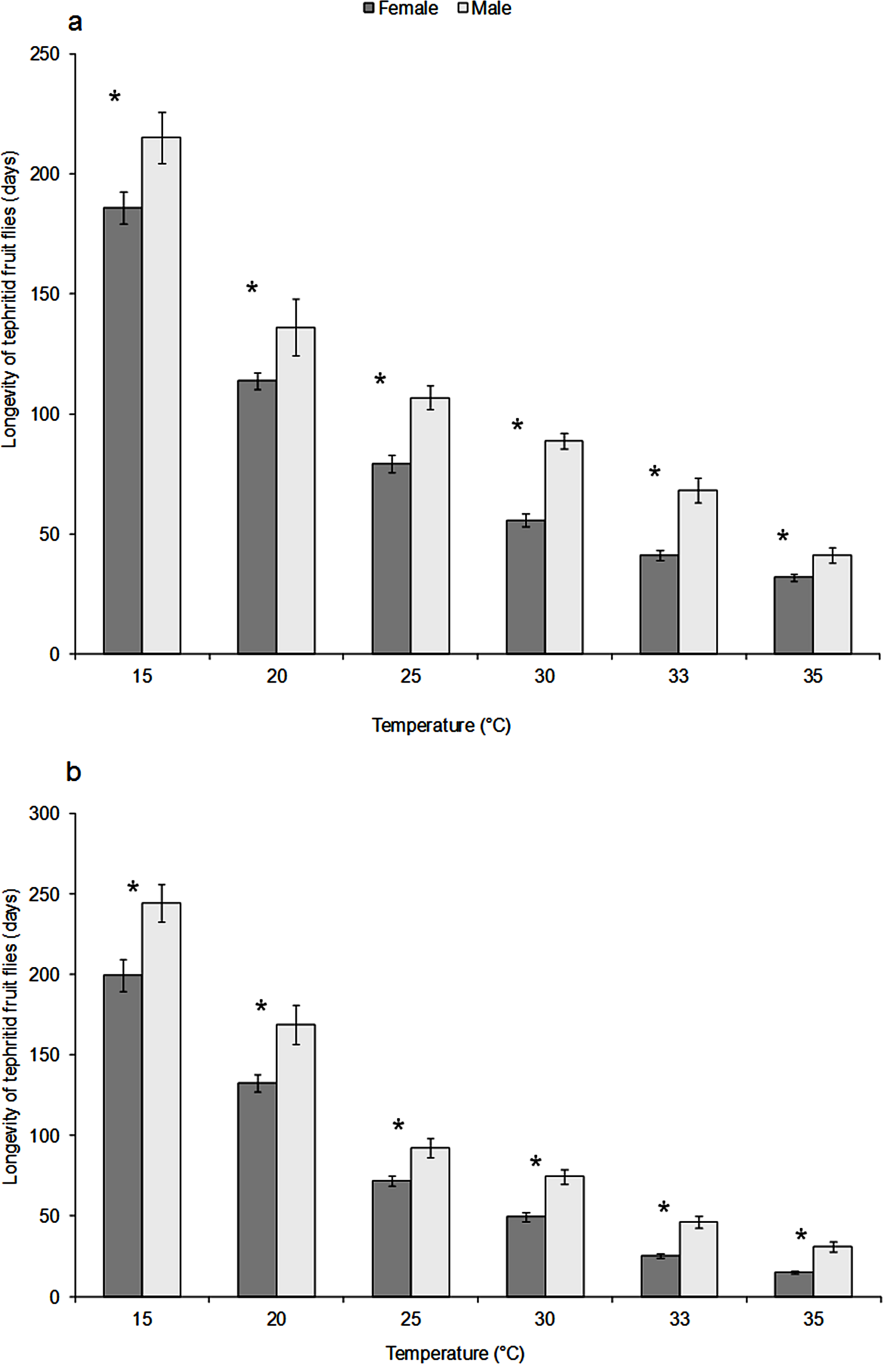
Mean longevity of females and males of *C*. *rosa* (3a) and *C*. *quilicii* (3b) at different temperature regimes. Asterisks above bars denote statistically significant differences comparing the sexes for each tested temperature category. Error bars represent the standard error generated from 15 replicates per condition.

The average longevity of adult females of *C*. *rosa* (F = 237.5; d.f. = 5, 84; P = 2x10^-14^) and *C*. *quilicii* (F = 202.9; d.f. = 5, 84; P = 2x10^-16^) were found to significantly decrease with increasing temperature (Fig 3A for *C*. *rosa* and Fig 3B for *C*. *quilicii*). The longevity of *C*. *rosa* was 31.9 ± 1.5 days at 35°C and 185.9 ± 6.7 days at 15°C, whereas that of *C*. *quilicii* was 15.0 ± 1.0 days at 35°C and 199.4 ± 10.0 days at 15°C. Within the same temperature regime, the longevity of female *C*. *rosa* and *C*. *quilicii* was significantly different at 20°C, 33°C and 35°C, whereas it was comparable at 15°C, 25°C and 30°C. There was significant interaction between the adult fruit fly species and temperature regimes for longevity (F = 5.75; d.f. = 5, 168; P = 6.35x10^-05^). Male longevity was greater than that of females for *C*. *rosa* (Fig 3A) and *C*. *quilicii* (Fig 3B) at all temperature regimes investigated.

**Fig 4:**
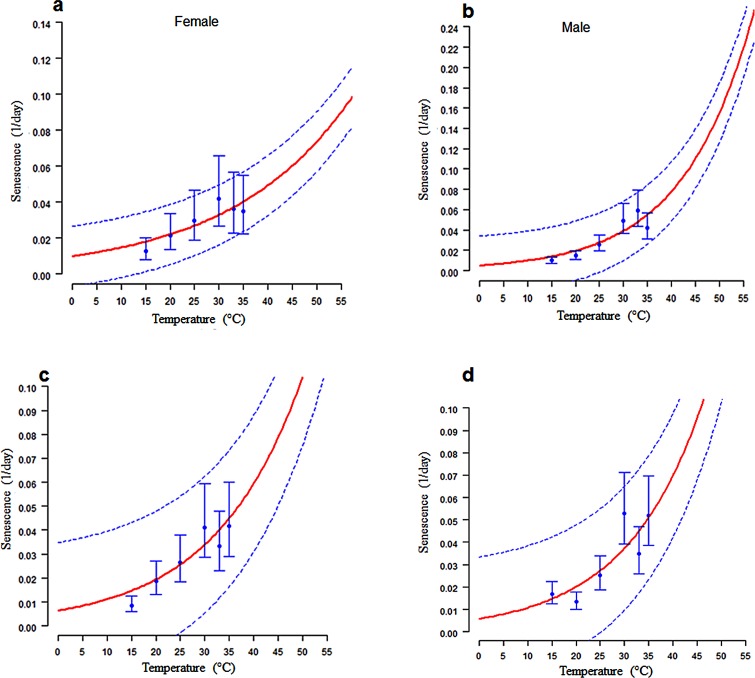
Temperature-dependent senescence rates (1/days) for adult *C*. *rosa* females (4a) and *C*. *rosa* males (4b); and for *C*. *quilicii* females (4c) and *C*. *quilicii* males (4d). Fitted curves: Exponential model for both sexes for each fruit fly species. The upper and lower 95% confidence intervals of the models are indicated. Bar represent standard deviation of the mean.

The exponential model gave a good fit to the observed mean senescence of both sexes of *C*. *rosa* (Fig 4A and Table 5) and *C*. *quilicii* (Fig 4B and Table 5).

**Table 5:** Estimated parameters (mean ± SE) of the exponential function: *r*(*T*) = *b*1.*exp*(*b*2.*T*); fitted to the mean senescence rates for adult life stages of *Ceratitis rosa* and *Ceratitis quilicii*.

	Life stage	Exponential function: *r*(*T*) = *b*1.*exp*(*b*2.*T*)					
		*b1*	*b2*	*r*^*2*^	*F*	*df*	*P*
*C*. *rosa*	Female	0.0098 (0.0041)	0.0403 (0.0137)	0.7470	11.8107	1,5	0.0264
	Male	0.0049 (0.0039)	0.0689 (0.0247)	0.7730	13.5979	1,5	0.0211
*C*. *quilicii*	Female	0.0061 (0.0029)	0.056 (0.0151)	0.8390	20.7951	1,5	0.0103
	Male	0.0058 (0.0042)	0.0624 (0.0234)	0.7280	10.7256	1,5	0.0306

Numbers in parentheses are standard errors of estimates

### Life table parameters of *C*. *rosa* and *C*. *quilicii*

**Fig 5:**
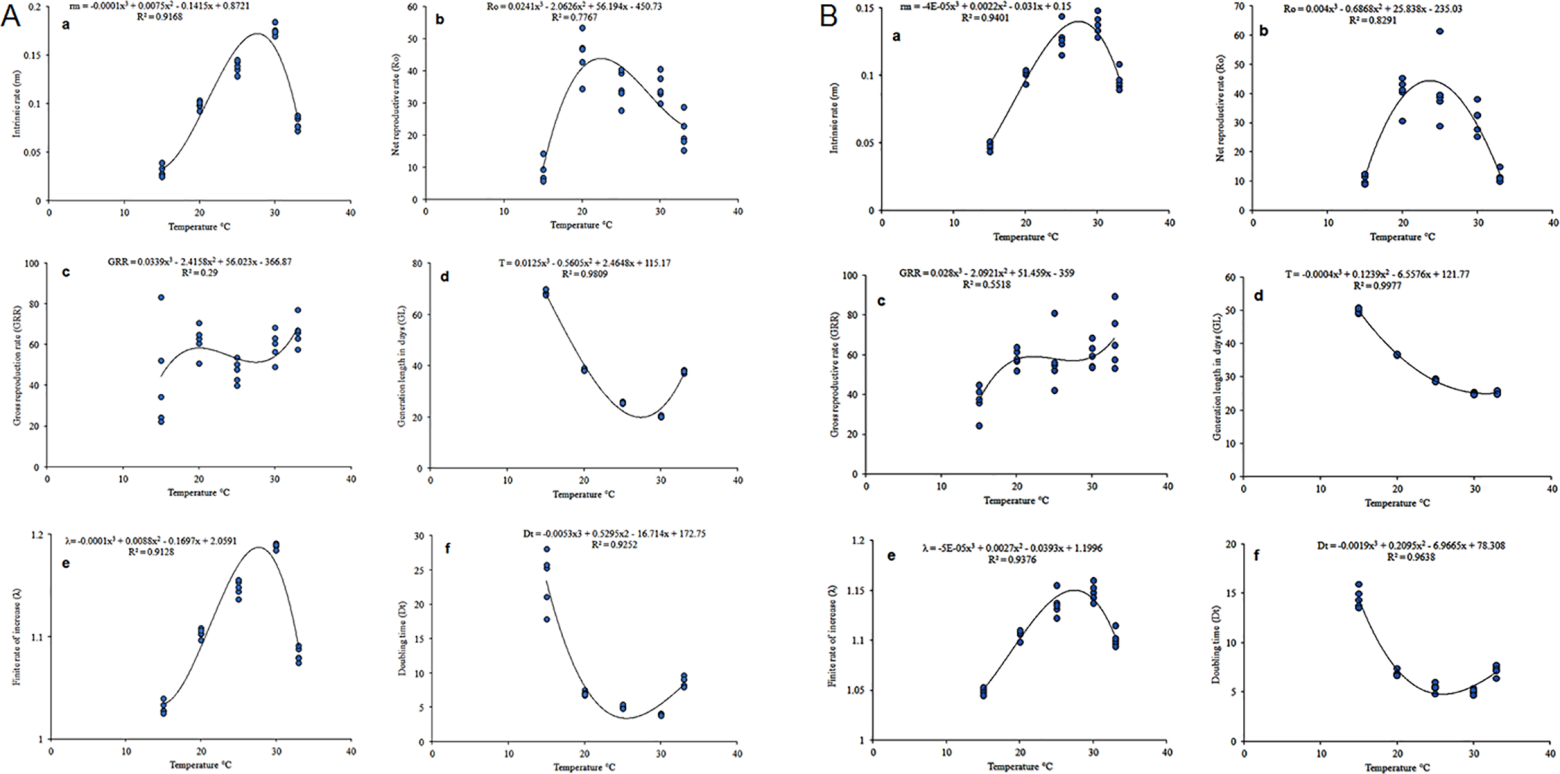
Life table parameters of *C*. *rosa* (**5a**) and *C*. *quilicii* (**5b**) estimated through model prediction over a range of six constant temperatures: Intrinsic rate of increase, r_m_; net reproduction rate, R_o_; gross reproductive rate, GRR; mean generation time, T; finite rate of increase, λ; and doubling time, D_t_.

The models established were compiled in an overall phenology model for stochastic simulation of *C*. *rosa* and *C*. *quilicii* to generate all the life table parameters (Table 6). The intrinsic rate of natural increase (r_m_), the net reproductive rate (R_o_), the finite rate of increase (λ), the mean generation time (Ί) and doubling time (D_t_) were estimated for each temperature regime. The life-table parameters showed that *C*. *quilicii* and *C*. *rosa* populations can develop successfully within the temperature range of 15–35°C. The highest intrinsic rate of increase of *C*. *rosa* was 0.19±2.7x10^-3^ at 30°C, while that of *C*. *quilicii* was 0.14±1.1x10^-3^ at 25°C. The net reproductive rate R_o_ differed significantly among temperatures with the highest at 30°C for *C*. *rosa* (189.17±5.49) and 25°C for *C*. *quilicii* (100.72±4.58) and. The lowest values of r_m_ and R_o_ for *C*. *rosa* and *C*. *quilicii* were recorded at 15°C and 35°C. Maximum values of finite rate of increase (λ) for *C*. *rosa* and *C*. *quilicii* recorded at 30°C. Values estimated for ‘T’ showed that the mean length of generations decreased with increase in temperatures from 84.57±0.58 days at 15°C to 35.47±0.28 at 33°C for *C*. *rosa*, where as that of *C*. *quilicii* varied from 83.70±0.57 days at 15°C to 36.96±1.06 at 35°C. The shortest doubling time for *C*. *rosa* and *C*. *quilicii* were observed at 30°C. Fitting of a polynomial model to the estimated life table parameters predicted temperatures between 25–30°C as the most favourable range for *C*. *rosa* (Fig 5A) and *C*. *quilicii* (Fig 5B) development, survival and reproduction.

**Table 6:** Life table parameters of *Ceratitis rosa* and *Ceratitis quilicii* estimated at different constant temperature regimes.

Parameters	Temperature (°C)											
	15		20		25		30		33		35	
	*C*. *rosa*	*C*. *quilicii*	*C*. *rosa*	*C*. *quilicii*	*C*. *rosa*	*C*. *quilicii*	*C*. *rosa*	*C*. *quilicii*	*C*. *rosa*	*C*. *quilicii*	*C*. *rosa*	*C*. *quilicii*
r_m_	0.04±3.1x10^-3e^A	0.04±4.4x10^-4e^A	0.07±7.7x10^-4d^A	0.08±8.2x10^-4d^A	0.14±1.1x10^-3b^A	0.12±9.2x10^-4b^A	0.19±2.7x10^-3a^A	0.11±1.7x10^-3a^A	0.10±2.3x10^-3c^A	0.09±2.5x10^-3c^A	0.04±2.0x10^-3e^A	0.04±7.4x10^-3e^A
R_o_	28.25±2.17^d^A	31.87±1.4^c^A	79.88±4.32^b^B	95.12±4.89^a^A	121.50±4.15^a^A	100.72±4.58^a^A	189.17±5.49^c^A	68.71±4.96^b^B	25.29±2.15^d^A	22.84±1.66^c^A	4.61±0.62^e^A	3.57±0.77^b^A
GRR	84.20±5.56^c^A	91.44±3.48^c^A	218.30±9.32 ^b^A	245.65±12.89 ^a^A	300.40±14.95 ^a^A	278.38±11.79 ^a^B	397.87±21.64 ^a^A	269.12±24.03^a^B	198.65±18.24 ^b^A	176.81±13.66 ^b^A	112.29±19.48 ^c^A	84.49±18.62 ^c^B
T	84.57±0.58^a^A	83.70±0.57^a^A	59.23±0.15^b^A	58.71±0.35^b^A	44.24±0.25^c^A	43.33±0.23^c^A	35.87±0.23^e^A	35.89±0.20^d^A	35.47±0.28^e^A	35.27±0.18^d^A	37.50±0.96^d^A	36.96±1.06^d^A
λ	1.04±1.1x10^-3e^A	1.04±4.6x10^-4c^A	1.08±8.3x10^-4d^A	1.08±8.8x10^-4b^A	1.12±1.2x10^-3b^A	1.11±1.0x10^-3a^A	1.14±3.0x10^-3a^A	1.12±1.9x10^-3a^A	1.11±2.5x10^-3c^A	1.09±2.7x10^-3b^A	1.05±8.5x10^-3e^A	1.02±1.3x10^-2d^A
D_t_	17.80±0.48^a^A	16.81±0.18^b^A	9.40±0.09^b^A	8.97±0.10^c^A	6.53±0.07^cd^A	6. 68±0.06^c^A	5.93±0.16^d^A	6.06±0.08^c^A	7.74±0.20^c^A	7.95±0.25^c^A	17.03±0.8^a^A	26.41±6.67^a^A

Means in the same column followed by the same upper case and in the same row followed by the same lower case letter are not significantly different [ANOVA and Student–Newman–Keul’s (SNK) test, P < 0.05]. r_m_, intrinsic rate of natural increase; Ro, net reproduction rate; GRR, gross reproduction rate; GT, mean generation time; λ, finite rate of increase; Dt, doubling time (days).

Adult female *C*. *rosa* and *C*. *quilicii* failed to reproduce at 37°C.

### Phenological model validation

**Fig 6:**
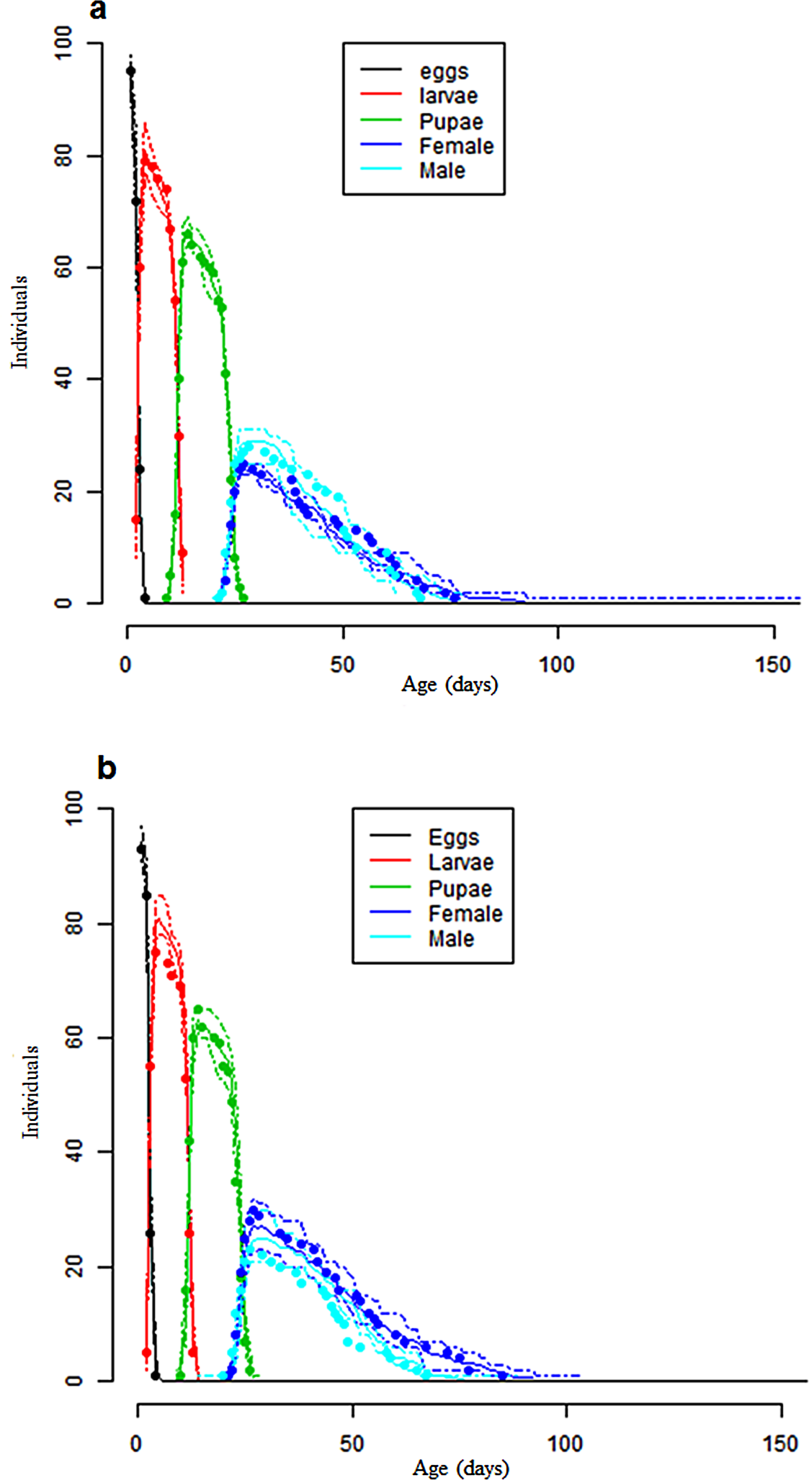
Model validation, observed and simulated life stage frequencies of *C*. *rosa* (6a) and *C*. *quilicii* (6b). Dots represent observed data points at fluctuating experiments, and the lines represent developmental frequencies simulated at fluctuated temepratures based on thermal reaction norms for constant temperatures. Solid lines are averages of the 50 simulations while the broken lines indicate minimum and maximum value obtained from the 50 simulations.

Model simulations with data collected under constant temperatures predicted good development duration and mortality in immature life stages when compared to the data collected under fluctuating temperatures for *C*. *rosa* and *C*. *quilicii* (Table 7 and Fig 6). Also, the simulated values of intrinsic rate of natural increase (r_m_), finite rate of population increase (λ) and doubling time (Dt) generated at constant temperature regimes were closely similar to the observed values (Table 8), making the developed phenology models capable of properly simulating the demographic parameters.

**Table 7:** Validation of the developed phenology model through comparison of observed and simulated developmental and mortality rates of *Ceratitis rosa* and *Ceratitis quilicii* life stages.

		Eggs		Larvae		Pupa		Euclidian distance	
		Developmental time (days)	Mortality (%)	Developmental time (days)	Mortality (%)	Developmental time (days)	Mortality (%)		
*C*. *rosa*	Simulated	2.325 ± 0.06	0.108 ± 0.06	9.105 ± 0.16	0.198 ± 0.06	11.873 ± 0.32	0.588 ± 0.18	Egg	6.40
	Observed	2.253 ± 0.15	0.151 ± 0.02	8.956 ± 0.10	0.139 ± 0.01	11.736 ± 0.12	0.662 ± 0.07	Larva	14.88
	P-value	0.0002	0.0045	0.0011	0.0005	0.0587	0.0657	Pupa	16.20
								Female	8.61
								Male	17.04
*C*. *quilicii*	Simulated	2.048 ± 0.11	0.102 ± 0.04	9.060 ± 0.12	0.165 ± 0.06	12.182 ± 0.16	0.594 ± 0.13	Egg	6.39
	Observed	2.098 ± 0.35	0.137 ± 0.21	9.100 ± 0.17	0.146 ± 0.09	12.019 ± 0.23	0.601 ± 0.19	Larva	10.99
	P-value	0.0404	0.0030	0.1552	0.1405	0.0006	0.8272	Pupa	8.89
								Female	9.18
								Male	11.12

Average ± SE: standard errors are calculated from the observed and simulated life table data

**Table 8:** Validation of the developed phenology model through comparison of observed and simulated population growth parameters of *Ceratitis rosa* and *Ceratitis quilicii*.

Population growth parameters						
Parameter	*C*. *rosa*			*C*. *quilicii*		
	Simulated	Observed	P-value	Simulated	Observed	P-value
r_m_	0.12 ± 0.02	0.108 ± 0.01	0.2350	0.095 ± 0.001	0.11 ± 0.01	0.0781
λ	1.09 ± 0.01	1.10 ± 0.01	0.1812	1.08 ± 0.02	1.10 ± 0.02	0.2125
Dt	10.74 ± 1.17	9.89 ± 0.45	0.3116	12.15 ± 2.18	12.03 ± 1.73	0.0975

Average ± SE: standard errors are calculated from the observed and simulated life table data

### Spatial mapping: Changes in *C*. *rosa* and *C*. *quilicii* distribution and abundance

**Fig 7:**
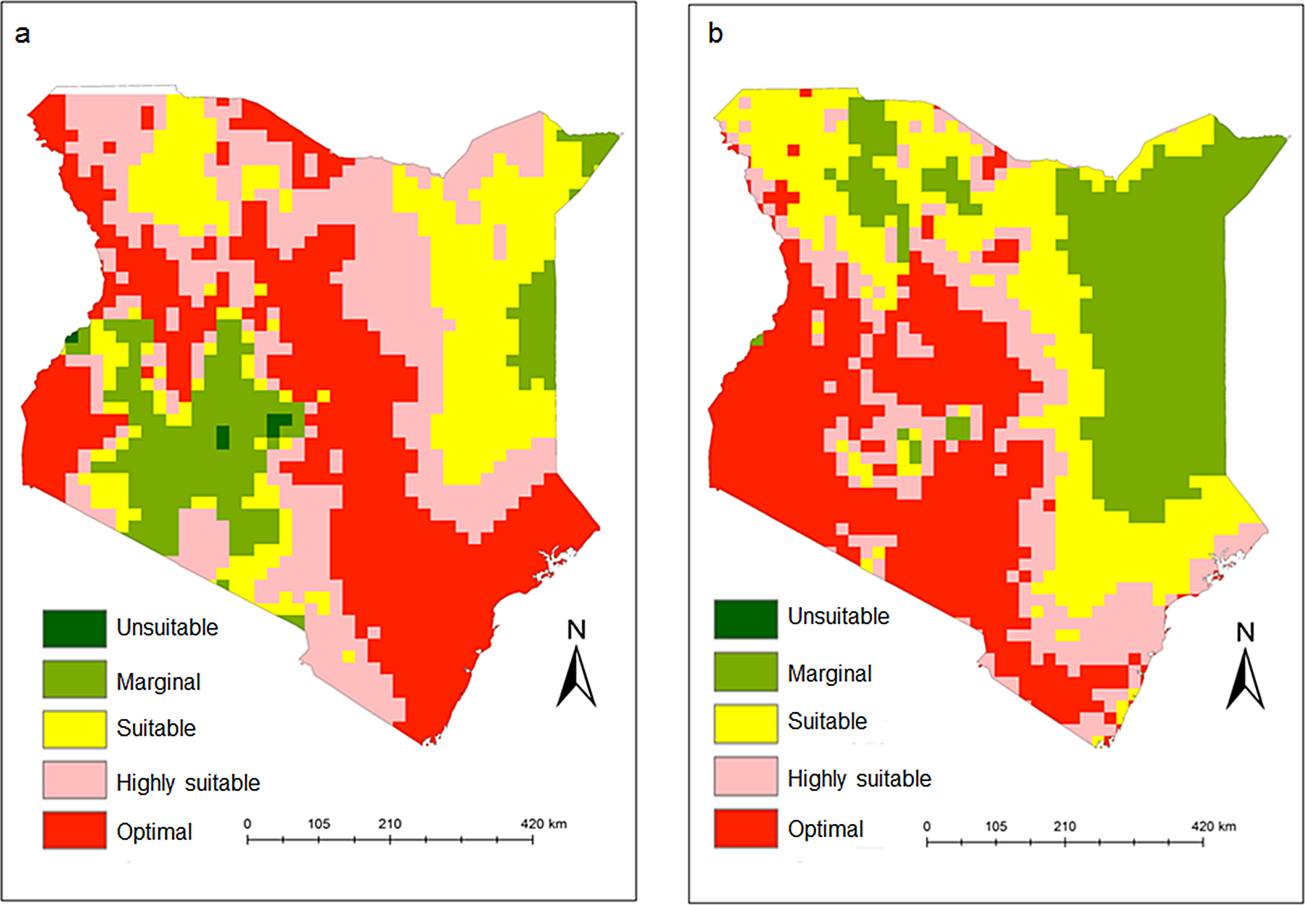
*Ceratitis rosa* (7a) and *C*. *quilicii* (7b) establishment risk index according to ILCYM model prediction in Kenya. Indices >0.6 is associated with potential permanent establishment. The ERI identifies the area in which the insect may survive and become established permanently.

**Fig 8:**
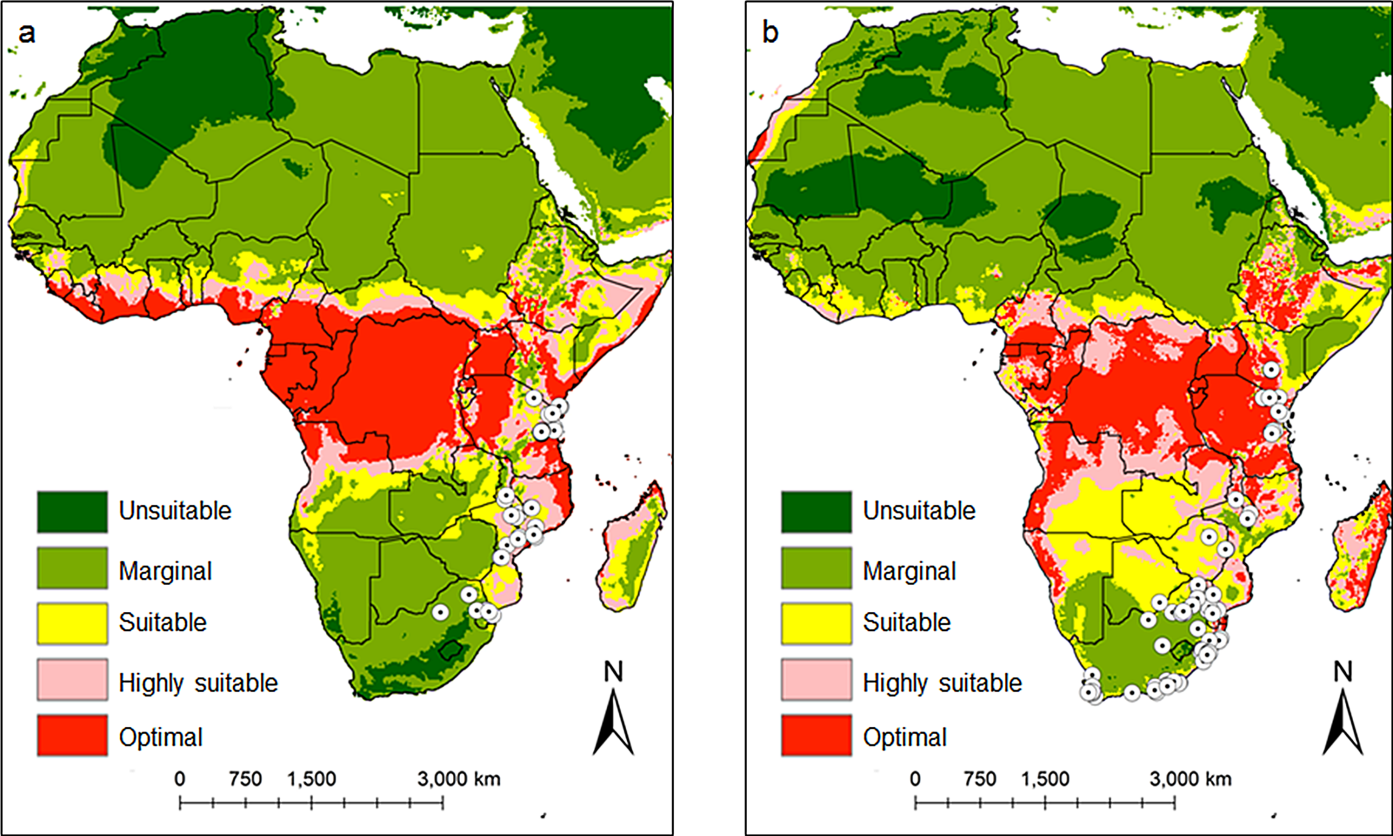
*Ceratitis rosa* (8a) and *C*. *quilicii* (8b) establishment risk index according to ILCYM model prediction in Africa, validated using known geo-referenced distribution data of each species. Indices > 0.6 are associated with potential permanent establishment. The ERI identifies the area in which the insect may survive and become established permanently.

In Kenya, the model predicted that *C*. *rosa* is well established along the coastal region, and Lake Victoria (Fig 7A) with ERI values ranging between 0.87 and 1.0, while *C*. *quilicii* is highly restricted along the highlands of Western and Central Kenya, especially around Lake Victoria (Fig 7B). Although, *C*. *rosa* and *C*. *quilicii* have different ecological requirements, the model predicted areas of sympatry between the two species in some regions in Africa (ERI between 0.7 and 0.87). Under the present climatic conditions, the establishment risk index reflects well the current distribution *C*. *rosa* (Fig 8A) and *C*. *quilicii* (Fig 8B) in Eastern and Southern Sub-Saharan African countries, which includes Kenya, Tanzania, Uganda, Rwanda, Somalia, Zambia, Botswana, Eritrea, Ethiopia, Mozambique, Swaziland and Republic of South Africa. The model also indicates higher ERI values for *C*. *rosa* and *C*. *quilicii* in several countries in West and Central Africa (Guinea-Bissau, Guinea, Sierra Leone, Libera, Côte d’Ivoire, Ghana, Togo, Benin, Nigera, Cameroon, Central African Republic etc), although neither species have been recorded in these countries. The model predictions for the potential establishment of *C*. *rosa* (Fig 8A) and *C*. *quilicii* (Fig 8B) was validated by using known geo-referenced distribution data (Fig 8A and 8B).

**Fig 9:**
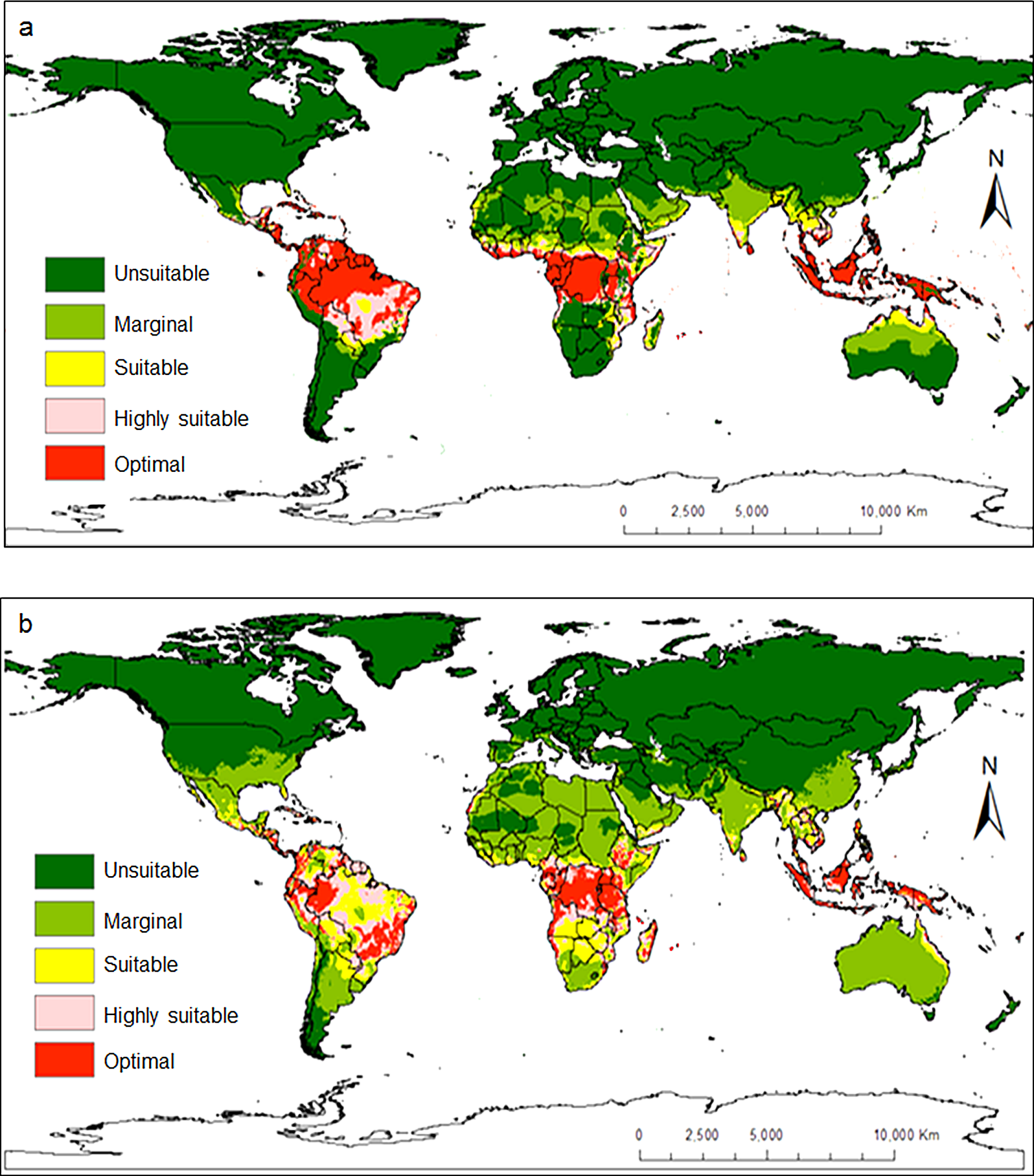
*Ceratitis rosa* (9a) and *C*. *quilicii* (9b) establishment risk index according to ILCYM model prediction in the world. Indices > 0.6 are associated with potential permanent establishment. The ERI identifies the area in which the insect may survive and become established permanently.

**Fig 10:**
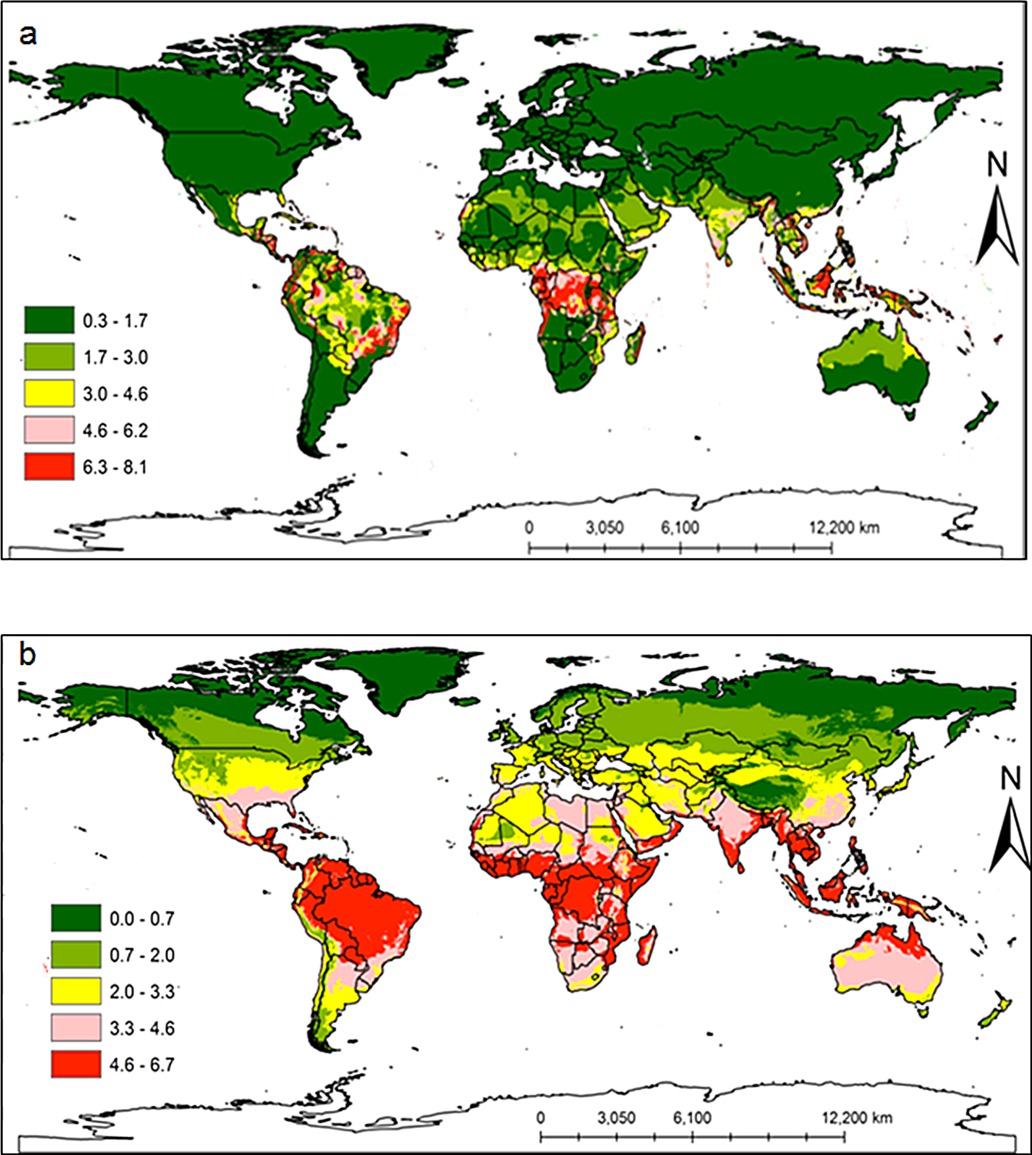
*Ceratitis rosa* (10a) and *C*. *quilicii* (10b) generation index (GI) according to ILCYM model predictions in the world. The GI estimates the mean number of generations that may be produced within a given year.

**Fig 11:**
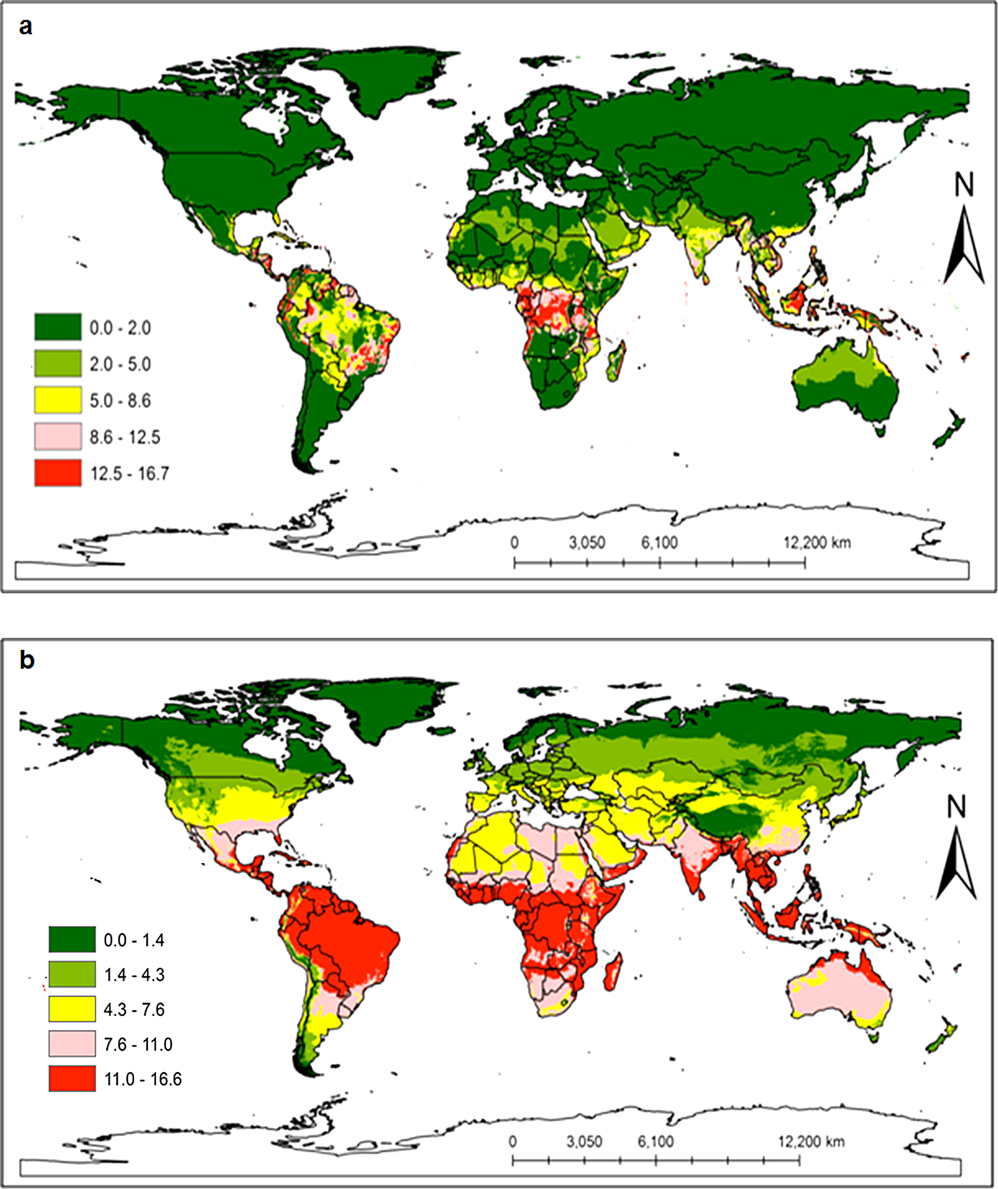
*Ceratitis rosa* (11a) and *C*. *quilicii* (11b) activity index (AI) according to ILCYM model predictions in the world. The AI takes the whole life history into consideration; an index value of 3 would illustrate a potential population increases by a factor of 1000 within one year.

Additional countries where neither pest has been reported but were found to be potentially suitable areas for invasion included: Madagascar, Middle East, Oceania, South and Southeastern Asia, Australia, Mexico, all Caribbean islands, Southern and Central America (Fig 9A and 9B for *C*. *rosa* and *C*. *quilicii*, respectively). The model also successfully predicted distinct broad potential ranges for *C*. *quilicii* in southern Europe and United States of America, which are not predicted to be suitable for *C*. *rosa*. The risk maps developed for the world with the climate data showed that areas with high-growth index (GI) (Fig 10A for *C*. *rosa* and Fig 10B for *C*. *quilicii*) also had high activity index (AI) (Fig 11A for *C*. *rosa* and Fig 11B for *C*. *quilicii***)**. However, it was found convenient to further evaluate the predictive ability of the model by estimating the potential number of generations of *C*. *rosa* (Fig 10A) and *C*. *quilicii* (Fig 10B), if they happen to invade and establish in different parts of around the world.

## Discussion

### Physiological effects of temperature

Our findings provide new evidence in support of the recent recognition that *C*. *rosa* comprises two distinct species, *C*. *rosa* and *C*. *quilicii* [61]. Whereas previous data was based on morphometrics, developmental physiology, cuticular hydrocarbons, pheromones and mating incompatibility studies, our study provides complementary evidence on distinct physiological differences between the two species in relation to temperature using climatic niche modelling. These findings agree with Mwatawala et al. [75] and Tanga et al. [3] who showed that the two species had different environmental requirements (lower developmental thresholds and development rates). The data confirms the hypothesis that *C*. *rosa* includes two separate entities with different ecological requirements [2]. This had been previously observed by Grout and Stoltz [2] and Duyck and Quilici [1], where the morphotype R1 (*C*. *rosa*) was dominant in lower altitude areas of South Africa whereas the morphotype R2 (*C*. *quilicii*) predominated in the high altitude area in Réunion [60]. The model also predicted areas of symphatry between the two species in the Mpumalanga and Kwa-Zulu Natal provinces of South Africa. The model was accurate and reliable in delineating climatic niche partitioning of *C*. *rosa* and *C*. *quilicii* and demonstrated that temperature remains one of the key factors that specifically determines the distribution range of both species. Our observation is further supported by the findings reported by Nyamukondiwa et al [76], which shows that plasticity in acute thermal tolerance might also be an important mechanism facilitating survival of fruit fly species upon introduction to novel thermal environments due to variation in their physiological responses particularly in cooler and more thermally variable geographical regions, which may contribute to their ongoing invasion success relative to other fruit fly species.

Using the second-order polynomial function to describe the relationship between mortality and temperature, another key finding in the present study was the lower larval developmental threshold for *C*. *quilicii* Kenyan population (4.8°C), which was consistent with that reported by Duyck and Quilici [1] for the population (3.1°C) in Réunion. This suggests that *C*. *quilicii* is better adapted to lower temperatures in comparison with *C*. *capitata* (10.2°C) and the native *C*. *catoirii* (8.9°C), a native species in Réunion. Although, the lower larval developmental threshold of *C*. *rosa* (9.6°C) was found to be similar to that of *C*. *capitata* and *C*. *catoirii* [1,2], *C*. *rosa* has been reported to out-competed the latter species [18–21, 23–25]. While it is not clear to what extent these species co-exist in the wild throughout their native range, it is likely that each species dominates in particular regions of the climate space. Indeed, Duyck et al. [56] have shown that climatic factors and niche segregration between *C*. *capitata* and *C*. *rosa* influence the distribution of these species in Réunion. However, caution may be required in interpreting these findings as other factors besides temperature also play major role in the national (local), regional and global invasion potential of insect species. For example, before invasion, major factors will include host preference and quality, relative humidity, tolerance of water stress, phenotypic plasticity, tourism and trade, climate change and land use, and after introduction dispersal can be influenced by high reproductive potential, rapid disperal ability and broad host range [15,55–57, 68–70,76,78,88,89,90].

This study presents for the first time the preoviposition, oviposition and post-oviposition period of *C*. *rosa* and *C*. *quilicii* across different temperature regimes. However, the developmental duration of the immature life stages of the two species did not differ substantially from that reported by Duyck and Quilici [1] and, Grout and Stoltz [2]. The longest preoviposition period of *C*. *rosa* (58 d) and *C*. *quilicii* (43 d) was recorded at 15°C, while the shortest pre-oviposition period of *C*. *rosa* (10 d) and *C*. *quilicii* (11 d) was observed at 35°C. This deviates from the findings of Duyck and Quilici [1], who reported that *C*. *rosa* will not produce mature eggs at 15 and 35°C. Although, we observed ovarian maturation at 15 and 35°C, the survival rate of the adult flies was very low, which explains the limited number of eggs laid at both temperature extremes. In the present study, *C*. *rosa* achieved its highest intrinsic rate of increase (0.19) and net reproductive rate (189) at 30°C, which is comparable to that of *B*. *dorsalis* at the same temperature (*r*_*m*_ = 0.174 and *R*_*o*_ = 196) but higher than that of *C*. *capitata* (*r*_*m*_ = 0.137 and *R*_*o*_ = 264.2) [77, 78]. On the other hand, *C*. *quilicii* exhibited higher intrinsic rate of increase (0.14) and net reproductive rate (100.7) at 25°C than *C*. *cosyra* (*r*_*m*_ = 0.109 and *R*_*o*_ = 96.6) and *B*. *dorsalis* (*r*_*m*_ = 0.128 and *R*_*o*_ = 30.5) [77].

The lifetime fecundity of *C*. *rosa* and *C*. *quilicii* reared within the temperature range of 20–30° was comparable to that of several fruit fly species including *C*. *capitata*, *B*. *dorsalis*, *B*. *zonata* and *Zeugodacus cucurbitatae* etc, which are the most widespread fruit fly pests of soft fruits and vegetables globally. Our results also showed that temperature-dependent mortality rates of *C*. *rosa* and *C*. *quilicii* was significantly higher at temperature extremes (lower and upper thermal threshold). Based on high temperature variation between the *C*. *rosa* and *C*. *quilicii*, our results suggest that *C*. *rosa* is physiologically more adapted to higher temperatures than *C*. *quilicii*, which explains the observed distribution of both species in the Eastern and Southern regions of Africa, with *C*. *quilicii* being more restricted to the highlands [58, 59], while *C*. *rosa* is restricted in the lowlands [50]. The cold tolerant nature of *C*. *quilicii* particularly raises major concern outside of Africa and its invasive powers require close attention. Overall, our observation agrees with the climatic variability hypothesis, which states that the thermal tolerance of an insect is directly proportional to the climate variability that the organism is exposed to [79].

### Prediction of the distribution of *C*. *rosa* and *C*. *quilicii*

Mapping the distribution of potentially invasive species is an iterative process. This is the first attempt at modelling the distribution of *C*. *rosa* and *C*. *quilicii* using temperature-dependent phenology models and projecting the risk of spread at national, regional and international level. Our models showed that *C*. *quilicii* will be more tolerant to a wider range of climatic conditions than *C*. *rosa*, occupying broader predicted regions than *C*. *rosa*. Results from the prediction showed that the current distribution and abundance of *C*. *rosa* and *C*. *quilicii* aligns with the occurrence data of both fruit fly species, which have been earlier reported from Eastern and Southern Africa [49,80,81]. Focusing on specific countries in the West and Central regions of Africa, we noted that these regions have a high risk of invasion and establishment of the pests and will threaten horticultural production in the region. Adequate phytosanitary management measures for fruits and vegetables proven to represent hosts of either species are recommended to curb the spread of both fruit fly species.

Risk maps also showed that the following countries and regions may be suitable for establishment of *C*. *rosa* and *C*. *quilicii*—Madagascar, the Middle East, Oceania, South and Southeast Asia, a large section of Australia, Mexico, all Caribbean islands, and Southern and Central America. The model also showed that *C*. *quilicii* has a broader distinct potential range of suitability in the southern regions of Europe and the United States of America (USA), but this was not the case for *C*. *rosa*. The area highly suitable for *C*. *rosa* in the USA was limited only to southern Florida. These findings are consistent with earlier studies by De Meyer et al. [10], who used correlative ecological niche modeling techniques based on genetic algorithm for rule-set prediction (GARP) and principal components analysis (PCA) to predict the global distribution of *C*. *rosa*. However, we believe that the incorporation of other known climatic drivers such as host plant diversity, natural enemy dynamics, relative humidity and rainfall that influence tephritid fruit fly species dynamics would enhance the validity of the predictions in the present studies [1,47,41,82,83, 88].

The global risk maps for both species showed that the areas with high-growth index (GI) also had high activity index (AI). The generation indices simulated for *C*. *quilicii* revealed that it was capable of completing 5–7 generations in a year, whereas *C*. *rosa* had 7–8 generations per year. These observations closely mirror the generation times that have been reported for several other tephritid fruit fly species [84–86].

Although the host range of *C*. *quilicii* has not been fully documented after having been proposed as a new species, we speculate that this might not be too different from those of *C*. *rosa* that also parallel those of *C*. *capitata* [34,87,80,1,10,3]. Together, these species represent a major threat to the horticulture industry in Africa and beyond, due to their capacity to become established and invade regions of the world outside their native range [10,81] based on the data presented here. Also, due to the significant differences in developmental rates of immatures, important population parameters such as the intrinsic rate of increase, survival and reproductive patterns of both *C*. *rosa* and *C*. *quilicii*, coupled with the lack of competitors and efficient natural enemies, and further compounded with the poor quarantine infrastructure in areas already invaded [18–21, 23–25], their invasion dynamics might be affected [88,89] such that the species turned to spread widely to new locations with far reaching social and economic consequences. Thus, this offers plausible justification to continue to assess their potential for establishment and spread.

The present study highlights the importance of temperature on the developmental rate, mortality, oviposition, fecundity and longevity of *C*. *rosa* and *C*. *quilicii*. Prediction of tephritid fruit fly pest phenology is an important component of developing a management strategy for potentially invasive pests. Overall, the proportion of the regions predicted to be highly suitable was found to be broader for *C*. *quilicii* than *C*. *rosa*, suggesting that *C*. *quilicii* may be tolerant to a wider range of climatic conditions than *C*. *rosa*. This raises major concerns for the global horticulture industry as both species tend to have high invasive powers and have the potential to out-compete other tephritid fruit flies [10]. These concerns are further compounded by low phytosanitary skills in the region. However, our findings provide important information to enhance monitoring and surveillance, and designing of local, regional and national-level phytosanitary and integrated pest management to curb the spread and potential establishment of both pest species across Africa and beyond. There is also a pressing need to carefully establish the host range of each species, and the susceptibility of fruits and vegetables at different stages of development when grown commercially for export.
